# Addition of anal encirclement to perineal proctosigmoidectomy: a retrospective review

**DOI:** 10.3389/fsurg.2025.1492690

**Published:** 2025-01-24

**Authors:** Aiya Amery, Kayla Marritt, Zarrukh Baig, Haven Roy, Dilip Gill, Nathan Ginther

**Affiliations:** ^1^ Department of Surgery, University of Saskatchewan, Saskatoon, SK, Canada; ^2^ Department of Surgery, University of British Columbia, Vancouver, BC, Canada

**Keywords:** perineal proctosigmoidectomy, Altemeier, Thiersch, anal encirclement, rectal prolapse

## Abstract

**Background:**

The optimal approach for the surgical management of rectal prolapse is individualized based on anatomical, functional, and surgical factors. In patients with significant comorbidities, perineal approaches are often preferred even though they are associated with higher recurrence rates compared to an abdominal approach. Although anal encirclement was one of the first procedures described for this condition, it is seldom employed given its high recurrence rates. There is currently a lack of data addressing a combination surgery, wherein both a perineal proctosigmoidectomy and anal encirclement are performed simultaneously.

**Aims:**

To evaluate the efficacy of combining perineal proctosigmoidectomy with anal encirclement using Nylon sutures compared to perineal proctosigmoidectomy alone.

**Methods:**

This was a single institution, non-randomized, retrospective study conducted at the Royal University Hospital, Saskatoon, Saskatchewan, Canada (July 2017 to October 2022). Patients over the age of 18 with full-thickness rectal prolapse who underwent either perineal proctosigmoidectomy alone or perineal proctosigmoidectomy with anal encirclement were included. There were 23 patients in the perineal proctosigmoidectomy group and 21 patients in the perineal proctosigmoidectomy with anal encirclement group. The primary outcome was prolapse recurrence. Secondary outcomes included operative time, length of hospital stay, and post-operative complications.

**Results:**

Patients who received perineal proctosigmoidectomy with anal encirclement had significantly lower rates of recurrent prolapse (9.5%) compared to perineal proctosigmoidectomy alone (34.8%) (*p* = 0.02). Patients who underwent the combined procedure had a shorter length of stay by 2.3 days (*p* = 0.03). There was no difference in post-operative complications or operating time.

**Conclusions:**

Routine anal encirclement in perineal proctosigmoidectomy reduces recurrence rates and length of stay without increasing operating time or complications.

## Introduction

Rectal prolapse is a benign but debilitating condition that significantly affects quality of life. It commonly affects those in their seventh decade of life and disproportionately affects women (1–7). It is defined as a circumferential, full-thickness protrusion of the rectal wall through the anal verge, often resulting in symptoms of incontinence, constipation, rectal bleeding, and psychological distress (1). Surgical intervention remains the definitive treatment for this condition, with the goals of preventing recurrence and improving continence (1–5, 8). Surgical management can be via transabdominal or perineal approaches, with many different techniques available, or definitively with an end colostomy. Although associated with higher recurrence, perineal approaches are generally preferred in elderly and high-risk patients (2, 9, 10).

Perineal proctosigmoidectomy, also known as the “Altemeier procedure”, is a perineal full-thickness resection of the prolapsed segment with a coloanal anastomosis (5, 9). This procedure avoids the physiological strain associated with a transabdominal approach and is often the preferred approach in elderly patients. Although our institution has reported considerably lower rates of recurrence in the past, historical data suggests that recurrence can be up to 58% and patients may also experience persistent incontinence with a perineal proctosigmoidectomy (1, 11). An alternative approach, often reserved for patients too frail to undergo bowel resection, is an anal encirclement procedure, also known as a “Thiersch procedure”. The procedure, first described in 1891, involved placing a silver wire around the anus (12). It is now more commonly performed with other materials such as a Nylon suture placed as a purse-string around the external sphincter to mechanically restrict the anus, preventing further prolapse and fecal incontinence (13–15). While anal encirclement is minimally invasive and can potentially be performed under local anesthetic alone, its recurrence rates are also up to 100% when performed without a proctosigmoidectomy (13).

Given the limitations of each procedure and the concerns with recurrence rates, surgeons at our institution started combining the two procedures 3 years ago in the hopes of improving outcomes. The rationale behind this combined approach is to reduce recurrence rates while maintaining the minimally invasive nature of a perineal approach. To our knowledge, there is only one prior study that has reported outcomes using a combination approach (16). Eftaiha et al. describe the use of a Bio-Thiersch mesh as an adjunct to perineal proctosigmoidectomy (16). Anal encirclement, however, is most commonly performed with a suture rather than a biological mesh implant (17). Here, we report the first study to evaluate the efficacy of routine anal encirclement with Nylon sutures in perineal proctosigmoidectomies. We hypothesize that combining these approaches will reduce recurrence rates compared to perineal proctosigmoidectomy alone.

## Methods

This was a retrospective analysis conducted at a tertiary care academic center (Royal University Hospital, Saskatoon, Canada) between July 2017 and October 2022. Informed consent was not required as the data was retrieved from our local database. The study was approved by the University of Saskatchewan Biomedical Research Board (BIO-3318). Patients over 18 years of age with full-thickness rectal prolapse who underwent an operation with either a perineal proctosigmoidectomy or proctosigmoidectomy with anal encirclement were included. Rectal prolapse was defined as a circumferential, full-thickness protrusion of the rectal wall through the anal verge. Recommendation for proctosigmoidectomy was made at the surgeon's judgment, taking into account comorbidities, surgical history, and performance status. There was no standardized protocol, and the decision was made between surgeon and patient after informed discussion.

Variables were analyzed using Pearson's Chi-squared Test or Mann-Whitney *U*-Test as appropriate. Repeated measurement ANOVA was used to compare changes. A *p*-value of <0.05 was considered significant. All data was analyzed using SPSS v.28 (IBM Corp., Armonk, NY).

Recurrence of rectal prolapse was the primary outcome measured. Secondary outcomes were operative time, length of hospital stay, and complications.

Recurrence was identified based on a confirmed physical examination by a colorectal surgeon. At the time of the study, all prolapse surgery in the province was being performed by three colorectal surgeons at a single institution. Recurrence was identified either at scheduled follow-up or by re-referral. Absence of re-referral was considered a surrogate marker for lack of recurrence, as patients were not routinely re-examined to exclude recurrence. Because all prolapse surgery in our province was conducted at the study site, we are confident that cases of recurrence were not missed due to referral to other surgeons. We used the STROBE cohort checklist when writing our report (18).

## Results

Twenty-three patients underwent a perineal proctosigmoidectomy (three of whom had a previous prolapse procedures, one Delorme, one Altemeier, and one abdominal rectopexy), and twenty-one patients underwent proctosigmoidectomy with encirclement (eight of whom had a prior perineal prolapse procedures, two Delorme and six Altemeiers). The two groups had similar baseline demographics, including age, BMI, gender, ASA score, and length of prolapse (Table 1).

**Table 1:** Baseline demographics for comparing proctosigmoidectomy (P) vs. proctosigmoidectomy with anal encirclement (P + A).

	P (*n* 23)		P + A (*n* 21)		*p* *
	Mean	SD	Mean	SD	
Age (*n*)	74		77		
33–42	2		1		*0* *.* *553*
51–90	18		19		
≥91	2		1		
Gender					
Female	20		20		*0*.*345*
Male	3		1		
BMI (kg/m^2^)	26.9	4.9	27.1	7.4	*0*.*526*
ASA (*n*)					
1	1		0		*0*.*468*
2	11		9		
3	11		12		
Length (cm)	9.4	5.5	8.6	2.4	*0*.*712*

P, proctosigmoidectomy; P + A, proctosigmoidectomy with anal encirclement.

* *p*-value is significant at *p* < 0.05.

As seen in Table 2, patients who received the combined procedure had a significantly lower rate of recurrence (9.5%) compared to the proctosigmoidectomy group alone (34.8%) (*p* = 0.02). The outcomes were superior despite more re-do cases in the combined group.

**Table 2:** Comparison of outcomes between proctosigmoidectomy (P) vs. proctosigmoidectomy with anal encirclement (P + A).

	P (*n* 23)		P + A (*n* 21)		*p*
	Mean	SD	Mean	SD	
Recurrence *n* (%)	8 (34.8%)		2 (9.5%)		*0*.023*
OR time (min)	53	20.5	57	22.7	*0*.*365*
Length of stay (days)	5.0	4.4	2.7	0.8	*0*.031*
Complications (*n*)	6		2		*0*.*101*

P, proctosigmoidectomy; P + A, proctosigmoidectomy with anal encirclement.

* *p*-value is significant at *p* < 0.05.

The average length of stay for patients who underwent the combined procedure was 2.3 days less (5.0 vs. 2.7, *p* = 0.03). There was no difference in the operating time between the two groups (53 vs. 57 min, *p* = 0.36). There were five complications in the proctosigmoidectomy group compared to two in the combined group, but this was not statistically significant (*p* = 0.10).

## Discussion

Our results demonstrate that the addition of anal encirclement to proctosigmoidectomy reduces recurrence by 25% and shortens hospital stay by 2.3 days without increasing operative time or complication rates. The high recurrence rates historically associated with anal encirclement alone or perineal proctosigmoidectomy alone have limited their use, but this combined approach can substantially improve outcomes.

Our results are validated by previous studies. The recurrence rates in the control group are similar to what our group has previously reported (5). Although these rates are considerably lower than the literature, they are consistent with our local rates, emphasizing the value of adding anal encirclements to perineal repairs (1, 2). In a previous study by Eftaiha et al., 62 patients with perineal proctosigmoidectomy were compared to 25 patients who received a proctosigmoidectomy with an adjunct Biomesh Thiersch (16). Recurrence rates were 29% in the control group and 8% in the combined group (*p* = 0.048), which are similar to our results of 34.8% (control group) and 9.5% (combined group). Our data provides additional evidence that the combination procedure improves outcomes. In contrast to Eftaiha et al., we chose a Nylon suture for encirclement rather than biomesh placement, as it is readily available, cost-effective, and avoids further dissection (16).

We also found that the average length of stay for patients who underwent the combined procedure was 2.3 days less (*p* = 0.03). One of the criteria for discharge after proctosigmoidectomy at our institution is the return of bowel function, and we speculate that anal encirclement may improve continence by strengthening the external sphincter. A shorter hospital stay improves healthcare costs and is particularly helpful for elderly patients who benefit from minimized hospitalization (19). However, the increase length of stay could also be attributed to the management of anastomotic leaks in the proctosigmoidectomy alone group. The lack of difference in operative times and post-operative complications between the two groups further emphasizes that the addition of anal encirclement does not incur additional stress to the patients or the healthcare system.

There were six complications in the proctosigmoidectomy group compared to two in the combined group which did not reach statistical significance (Table 2). Three of the six complications in the proctosigmoidectomy group were anastomotic leaks. One leak was managed conservatively with IV antibiotics. The other two required take back to the operating room for a washout and formation of end colostomy. No patients were treated by proximal diversion while leaving the anastomosis *in situ*. In the combined group there was one leak, which was attributed to perforation at the posterior anastomotic site of a prior proctosigmoidectomy. We can only hypothesize as to the reason for increased leak rate in the proctosigmoidectomy alone group; the numbers are small, and it is difficult to draw definite conclusions. 2 of the 3 leaks were operated upon by the same surgeon, which raises the possibility of different surgical technique. However, it is important to consider that all patients were over the age of 85 and the increased frequency of leaks could be due to age-related factors, including diminished physiological reserves and impaired healing capacity inherent in this population. Future prospective studies could include the use of butylcholinesterase enzymes as a predictive factor for leak rates (21). Other complications in both groups included pelvic hematomas and urinary retention. Only one patient in the control group described incontinence at follow-up, and none in the combination group. Incontinence was assessed clinically pre and postoperatively. We did not identify any complications related to the encirclement itself in the combination group. There were no cases of suture protrusion or erosion identified.

Although patients in our study were not randomized, both groups were similar in baseline characteristics (average age, sex, BMI, ASA, and length of prolapse). These characteristics are also reflective of the general population that presents with prolapse (6). An interesting finding in our study was that there were more patients with recurrences in the intervention group, which would bias the results against the combined approach as previous prolapse is a risk factor for recurrence (17, 20). In fact, our study found that of the twenty-one patients who underwent a combined procedure, eight had a previous prolapse procedure and only one recurred (1/8). In the proctosigmoidectomy group, three patients had previously undergone a procedure for rectal prolapse and all three recurred (3/3).

This study has several limitations. The retrospective design, combined with the non-randomized small sample size, can lead to potential selection bias and limit generalizability. Another limitation of this study was that the time to recurrence was not measured, which could have provided nuanced insights into the long-term successes of the combined procedure. In the Swedish Rectal Prolapse Trial, 20% of recurrences occurred later than three years after surgery (8). We started performing routine anal encirclements only 3 years ago, and only 40% of patients in the control group had their operation more than three years ago, hence the 3-year follow-up. Finally, patients in our study were not routinely re-examined at the 3-year follow-up to rule out recurrence. Given the vast geography of our catchment area and the logistical challenges involved in transporting frail patients, we chose not to request in-person assessments. Instead, we used a lack of re-referral to confirm operative success at the 3-year mark. Future research should focus on larger, multi-center, randomized controlled trials to validate these findings.

## Conclusion

In our opinion, the addition of routine anal encirclement with a Nylon suture is easy to learn and straightforward to perform. The participating surgeons taught themselves how to perform it by reviewing the literature and procedural videos. This additional maneuver can significantly reduce recurrence rates without adding any additional operative time, complications, or healthcare costs in high-risk patients with rectal prolapse.

## Data Availability

The raw data supporting the conclusions of this article will be made available by the authors, without undue reservation.

## References

[1] Bordeianou L Paquette I Johnson E Holubar SD Gaertner W Feingold DL Clinical practice guidelines for the treatment of rectal prolapse. Dis Colon Rectum. (2017) 60:1121–31. 10.1097/DCR.0000000000000889 28991074

[2] Madsen M. Perineal approaches to rectal prolapse. Clin Colon Rectal Surg. (2008) 21:100–5. 10.1055/s-2008-1075858 20011405 PMC2780197

[3] Senapati A Gray RG Middleton LJ Harding J Hills RK Armitage NCM PROSPER: a randomised comparison of surgical treatments for rectal prolapse. Colorectal Dis. (2013) 15:858–68. 10.1111/codi.12177 23461778

[4] Shin EJ. Surgical treatment of rectal prolapse. J Korean Soc Coloproctol. (2011) 27:5. 10.3393/jksc.2011.27.1.5 21431090 PMC3053504

[5] Roy HM Baig Z Karimuddin AA Raval MJ Brown CJ Phang PT A comparison of perineal stapled prolapse resection and the altemeier procedure at 2 Canadian academic hospitals. Can J Surg. (2023) 66:E8–12. 10.1503/cjs.008421 36596586 PMC9829033

[6] Gurland B Zutshi M. “Rectal Prolapse”. The ASCRS Manual of Colon and Rectal Surgery. Cham: Springer International Publishing (2019). p. 777–81. 10.1007/978-3-030-01165-9_60

[7] Boccasanta P Venturi M Agradi S Calabrò G Bordoni L Missaglia C Is it possible to reduce recurrences after altemeier’s procedure for complete rectal prolapse? Twenty-year experience in 130 consecutive patients. Langenbecks Arch Surg. (2021) 406:1591–8. 10.1007/s00423-021-02091-2 33538872

[8] Smedberg J Graf W Pekkari K Hjern F. Comparison of four surgical approaches for rectal prolapse: multicentre randomized clinical trial. BJS Open. (2022) 6(1):zrab140. 10.1093/bjsopen/zrab140 35045155 PMC8769527

[9] Cirocco WC. The Altemeier procedure for rectal prolapse: an operation for all ages. Dis Colon Rectum. (2010) 53:1618–23. 10.1007/DCR.0b013e3181f22cef 21178855

[10] Altomare DF Binda G Ganio E De Nardi P Giamundo P Pescatori M. Long-term outcome of Altemeier’s procedure for rectal prolapse. Dis Colon Rectum. (2009) 52:698–703. 10.1007/DCR.0b013e31819ecffe 19404077

[11] Schabl L Hull T Ban K Steele SR Spivak A. Recurrence rates and risk factors in Altemeier procedure for rectal prolapse: a multicenter study. Dis Colon Rectum. (2024) 67(11):1465–74. 10.1097/DCR.0000000000003439 39087690

[12] White M. Operation for rectal prolapse using stainless steel ring. Lancet. (1961) 277:858–9. 10.1016/S0140-6736(61)90179-9 13784839

[13] Baker WNW. Results of using monofilament nylon in Thiersch’s operation for rectal prolapse. Br J Surg. (1970) 57(1):37–9. 10.1002/bjs.1800570108 5411586

[14] Poole G V Pennell TC Myers RT Hightower F. Modified Thiersch operation for rectal prolapse. Technique and results. Am Surg. (1985) 51:226–9. 3157338

[15] Haskell B Rovner H. A modified Thiersch operation for complete rectal prolapse using a Teflon prosthesis. Dis Colon Rectum. (1963) 6:192–5. 10.1007/BF02617326 13960877

[16] Eftaiha SM Calata JF Sugrue JJ Marecik SJ Prasad LM Mellgren A Bio-Thiersch as an adjunct to perineal proctectomy reduces rates of recurrent rectal prolapse. Dis Colon Rectum. (2017) 60:187–93. 10.1097/DCR.0000000000000723 28059915

[17] Warwick A Zimmermann E Boorman P Smart N Gee A. Recurrence rate after Delorme’s procedure with simultaneous placement of a Thiersch suture. Ann R Coll Surg Engl. (2016) 98:419–21. 10.1308/rcsann.2016.0148 27092405 PMC5209973

[18] von Elm E Altman DG Egger M Pocock SJ Gøtzsche PC Vandenbroucke JP. The strengthening the reporting of observational studies in epidemiology (STROBE) statement: guidelines for reporting observational studies. Lancet. (2007) 370:1453–7. 10.1016/S0140-6736(07)61602-X 18064739

[19] Thillainadesan J Yumol MF Suen M Hilmer S Naganathan V. Enhanced recovery after surgery in older adults undergoing colorectal surgery: a systematic review and meta-analysis of randomized controlled trials. Dis Colon Rectum. (2021) 64:1020–8. 10.1097/DCR.0000000000002128 34214055

[20] Nugent E Spivak A Gurland BH Shawki S Hull TL Zutshi M. Does the length of the prolapsed rectum impact outcome of surgical repair? Dis Colon Rectum. (2021) 64:601–8. 10.1097/DCR.0000000000001856 33463998

[21] Verras GI Mulita F. Butyrylcholinesterase levels correlate with surgical site infection risk and severity after colorectal surgery: a prospective single-center study. Front Surg. (2024) 11:1379410. 10.3389/fsurg.2024.1379410 39229253 PMC11368738

[22] Amery A Ginther N Gill D. Addition of Anal Encirclement to Perineal Proctosigmoidectomy: A Retrospective Review. the Society for Surgery of the Alimentary Tract. Chicago: Gastroentrology (2023). 10.3389/fsurg.2025.1492690 PMC11802564 39926041

